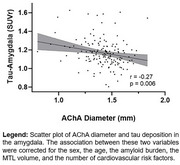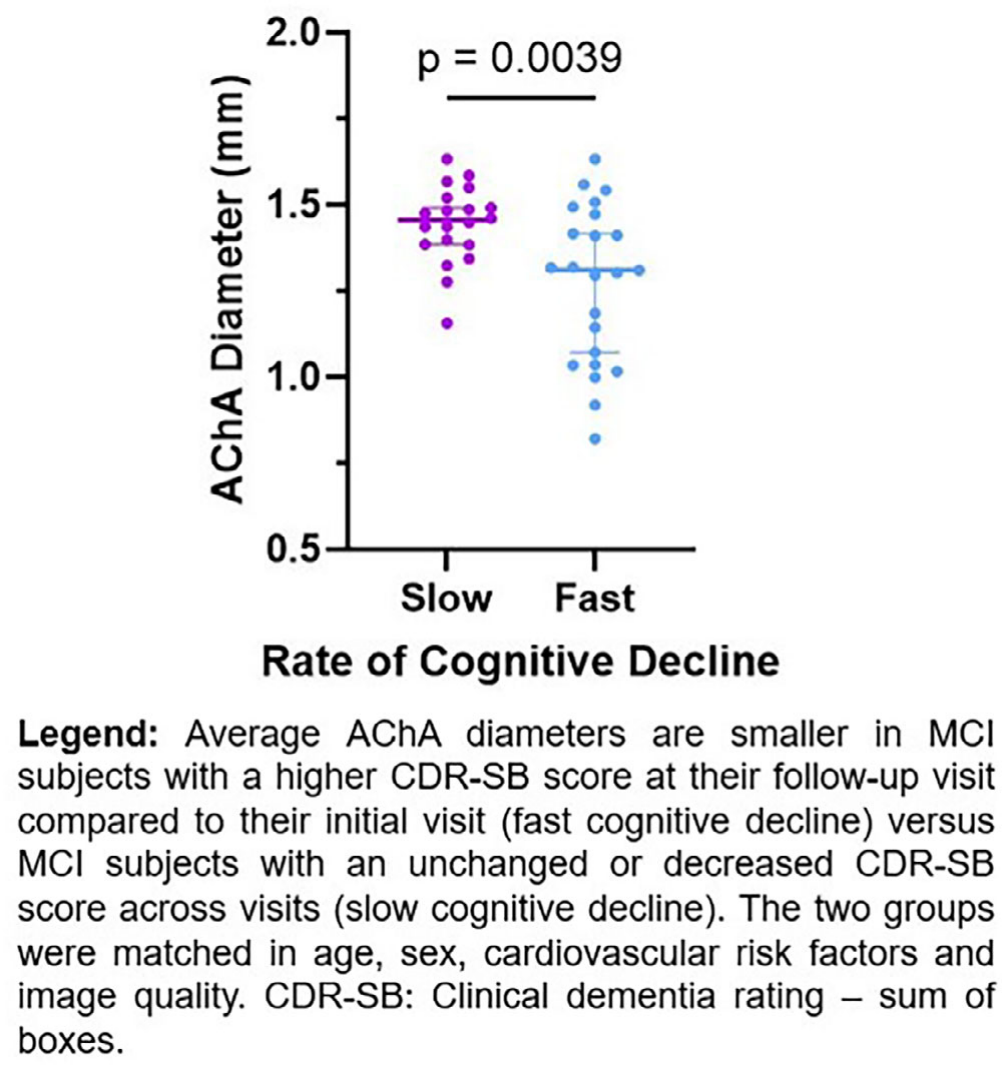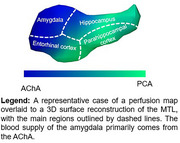# A vascular component to the selective vulnerability of the medial temporal lobe in Alzheimer’s disease

**DOI:** 10.1002/alz70862_110824

**Published:** 2025-12-23

**Authors:** Vincent Doyon, Félix Janelle, Samantha Côté, Monica Sean, Davy Vanderweyen, Julia Huck, Christian Bocti, Pascal Tétreault, Kevin Whittingstall

**Affiliations:** ^1^ Université de Sherbrooke, Sherbrooke, QC Canada; ^2^ Université de Montréal, Montréal, QC Canada

## Abstract

**Background:**

While several lines of evidence suggest that pathological tau propagates in the brain in a prion‐like manner via the synapses, little is known about the reason for its initial appearance in the medial temporal lobe (MTL). Many studies have reported that vascular health markers such as cerebral blood flow and arterial narrowing are associated with tau burden. Moreover, because abnormal tau deposition can arise from ischemic lesions, it is possible that initial tau aggregation in the Alzheimer’s disease (AD) continuum is driven by vascular narrowing that causes chronic hypoperfusion. Herein, we tested the hypothesis that vascular narrowing of MTL arteries is associated with tau levels and cognitive decline.

**Method:**

In a healthy cohort from the OASIS dataset, we analyzed the relationship between tau burden (positron emission tomography) and arterial size (time‐of‐flight magnetic resonance imaging) of MTL‐supplying arteries, namely the anterior choroidal artery (AChA) and the posterior cerebral artery (PCA). Then, we evaluated if arterial diameter was associated with deterioration of cognition in a second cohort diagnosed with mild cognitive impairment (MCI). Finally, in a cohort of 5 young and healthy participants, we sought to determine the pattern of perfusion of the AChA and the PCA to the MTL using the super‐selective arterial spin labelling sequence (SS‐ASL) in magnetic resonance imaging.

**Result:**

Analyses from the OASIS dataset reveal that the AChA lumen diameter correlated negatively with tau levels in the amygdala. In our MCI cohort, the AChA was the only Circle of Willis artery whose diameter was associated with the progression of cognitive decline. The SS‐ASL data suggest that the blood supply of the amygdala is almost entirely dependent on the AChA, which agrees with the idea that AChA narrowing would primarily impact tau levels in that region.

**Conclusion:**

This study provides the first evidence of an artery‐specific effect of vascular narrowing on key features of AD. It suggests that vascular health of the AChA might be critical in halting the progression of AD and that selective vulnerability of the MTL to tau pathology partly emerges from a compromised AChA.